# Direct and Indirect Protection with Pediatric Quadrivalent Live-Attenuated Influenza Vaccination in Europe Estimated by a Dynamic Transmission Model

**DOI:** 10.36469/9801

**Published:** 2017-07-28

**Authors:** Laetitia Gerlier, Judith Hackett, Richard Lawson, Sofia Dos Santos Mendes, Catherine Weil-Olivier, Markus Schwehm, Martin Eichner

**Affiliations:** 1 QuintilesIMS, Real-World Evidence Solutions, Zaventem, Belgium; 2 AstraZeneca, Gaithersburg, MD, USA; 3 AstraZeneca, Brussels, Belgium (Current affiliation: MSD, Brussels, Belgium); 4 Department of Pediatrics University Paris VII, Paris, France; 5 ExploSYS GmbH, Leinfelden-Echterdingen, Germany; 6 Institute for Clinical Epidemiology and Applied Biometry University of Tübingen, Tübingen and 7Epimos GmbH, Dusslingen, Germany

**Keywords:** europe, dynamic transmission model, indirect protection, live-attenuated influenza vaccine, pediatric vaccination, seasonal influenza

## Abstract

**Objectives:** To estimate the public health impact of annual vaccination of children with a quadrivalent live-attenuated influenza vaccine (QLAIV) across Europe.

**Methods:** A deterministic, age-structured, dynamic model was used to simulate influenza transmission across 14 European countries, comparing current vaccination coverage using a quadrivalent inactivated vaccine (QIV) to a scenario whereby vaccination coverage was extended to 50% of 2–17 year-old children, using QLAIV. Differential equations described demographic changes, exposure to infectious individuals, recovery and immunity dynamics. For each country, the basic reproduction number (R0) was calibrated to published influenza incidence statistics. Assumed vaccine efficacy for children was 80% (QLAIV) and 59% (QIV). Symptomatic cases cumulated over 10 years were calculated per 100 000 person-years. One-way sensitivity analyses were conducted on QLAIV efficacy in 7–17 year-olds (59% instead of 80%), durations of natural (±3 years; base case: 6, 12 years for influenza A, B respectively) and QLAIV vaccine-induced immunity (100% immunity loss after 1 season; base case: 30%), and R0 (+/-10% around all-year average value).

**Results:** Across countries, annual QLAIV vaccination additionally prevents 1366–3604 symptomatic cases per 100 000 population (average 2495 /100 000, ie, a reduction of 47.6% of the cases which occur in the reference scenario with QIV vaccination only). Among children (2–17 years), QLAIV prevents 551–1555 cases per 100 000 population (average 990 /100 000, ie, 67.2% of current cases). Among adults, QLAIV indirectly prevents 726-2047 cases per 100 000 population (average 1466 /100 000, ie, 40.0% of current cases). The most impactful drivers of total protection were duration of natural immunity against influenza A, R0 and QLAIV immunity duration and efficacy. In all evaluated scenarios, there was a large direct and even larger indirect protection compared with the reference scenario.

**Conclusions:** The model highlights direct and indirect protection benefits when vaccinating healthy children with QLAIV in Europe, across a range of demographic structures, contact patterns and vaccination coverage rates.

## INTRODUCTION

Published in 2012, the World Health Organization (WHO)'s position paper on influenza vaccination indicates that children less than 5 years of age (and especially those aged less than 2 years) bear a high burden of influenza, and ought to be targeted for vaccination where resources are available.[1] Children also play an important role in the transmission of influenza viruses in the community.[2] Therefore, besides protecting them directly, pediatric influenza vaccination further aims at reducing the overall spread of the virus and, thus, at indirectly reducing the number of cases in the entire population, particularly in those at high risk of developing complications. In most European Union (EU) countries however, influenza vaccination policies target only individuals with high complication risk from 6 months of age, ie, with chronic disease/immune deficiency or aged ≥65 years, representing about 180 million individuals (36%) in the EU-27 population.[3]

Countries having implemented influenza vaccination programmes including healthy children now have data available that show the real-life benefits of such vaccination strategies. Indeed, reported studies document the indirect (herd) protection effect when vaccinating school-age children and the direct effect of routine pediatric vaccination programmes.[4-7] In ddition to real-life studies, the potential added value of using mathematical models to assess and compare the impact of alternative vaccination strategies is appreciable.[8-10] In our study, we used a dynamic transmission model (initially developed for Germany[11,12] and previously adapted to France[13] and Belgium[14]) to estimate the public health impact of pediatric influenza vaccination in different European countries.

### Aims and Objectives

This study aims to estimate the public health impact of extending annual influenza vaccination from high-risk individuals to include healthy children aged 2–17 years, in 14 European countries, comprising Austria, Belgium, Finland, France, Germany, Greece, Italy, Luxembourg, The Netherlands, Poland, Portugal, Spain, Sweden, and the United Kingdom.

## METHODS

### Study Design

A deterministic, age-structured, dynamic transmission model was used to simulate the transmission of influenza in the population and to compare different vaccination strategies including direct and indirect protection effects. Demographic changes and transmission dynamics are described by a system of interacting differential equations. Technical details on the two-strain version of the simulation tool, previously used for Germany, were published elsewhere.[11,12] The current simulation tool considers the concomitant and independent transmission of four influenza strains: A(H1N1), A(H3N2), one B strain coming from the B/Victoria lineage, and one B strain coming from the B/Yamagata lineage. Model inputs are presented in Table 1 (values common to all countries) and Table 2 (country-specific values).

### Demographics and Contact Patterns

The population was subdivided into 1-year age cohorts and risk classes. Demographic data and population projections for each country were retrieved from EuroStat and from the national statistics institute of each country (Table 2). In line with the current recommendations in most EU countries, the "high-risk" group includes all individuals aged >65 years, and individuals from 6 months of age with immunodeficiency or any chronic cardiovascular, hepatic, renal, metabolic, neurological, or pulmonary comorbidity.[15] In 2014, it was estimated that 36% of the European population had at least one risk factor.[3] For the model, we further assumed that the "high-risk" prevalence was 16.1% until the age of 44 years and 32.1% for the age group 45–64 years, plus, by definition, all persons aged >65 years (Table 1).[3,15] "Non high-risk" individuals are referred to as "low-risk". Contact patterns between individuals (i.e. average age-dependent numbers of contacts per person per day) were derived from the European Polymod study, using the matrix for physical and non-physical contacts.[16] Contact data from a neighbouring country was used in absence of country-specific information (Table 2).

### Natural History of Influenza

The all-year average of the seasonally fluctuating basic reproduction number $R_0$ was calibrated to country-specific reported incidence data for laboratory-confirmed influenza. Calibration targets for influenza were derived from available incidence data of each country: either infection incidence,[17,18] symptomatic cases,[19-22] or physician visits,[23-26] averaged over two or more seasons (Table 2). The basic reproduction number $R_0$, representing the number of secondary infections produced by a single infected case, was assumed to vary over the year: it was 43% higher than the all-year average around Christmas and 43% lower in summer.[27] The same value of $R_0$ was used for each one of the four influenza strains which were assumed to be transmitted independently. To avoid virus transmission becoming extinct in the summer, the whole population was assumed to be further exposed to an external infection rate of 1 per 1000 susceptible person-years, which also fluctuated seasonally. The average duration of latency in the model was 1 day, followed by an average 5-day period of contagiousness.[28] Following infection, natural immunity was assumed to last on average for 6 years for influenza A and 12 years for influenza B.[27] The proportion of individuals developing symptoms in case of infection was assumed to be 66.9% (Table 1).[29]

### Compared Vaccination Strategies

After immunity patterns of the simulated population had been initialized during 20 years using the observed vaccination coverage and vaccine composition, allowing for transmission of the four influenza strains, two strategies were compared during ten influenza seasons, starting 2015–2016: (1) the reference strategy was the current coverage of high-risk individuals using quadrivalent inactivated influenza vaccine (QIV); (2) the evaluated strategy was an extension of current vaccination policy to 2–17 year-old healthy children using an intranasal, quadrivalent live-attenuated influenza vaccine (QLAIV) and increasing the coverage from the current level to a final coverage of 50% achieved in three annual steps. Children suffering from a severe form of asthma, representing about 11% of all high-risk children,[30] are not eligible for a live-attenuated vaccine and, thus, continued to receive inactivated vaccine (QIV) in the model. Current vaccination coverage rates per age-risk group and country were derived from the most recent reports by the European Centre for Disease Prevention and Control (ECDC),[31,32] multi-country surveys,[33,34] and country-specific studies[9,35-48] (Table 2). Vaccinations were assumed to be performed annually from October 1 to November 30. According to a study by the French sick fund,[49] individuals vaccinated in a given year had a higher probability of being re-vaccinated the following year (odds ratio 30–60). A preferential re-vaccination factor was implemented in the simulations accordingly (Table 1).

### Vaccination Properties

In the model, the vaccine efficacy was considered globally against all influenza strains. The vaccine efficacy against influenza infection in children aged 2–17 years, assessed in meta-analyses, was 59% (95% confidence interval [41–71%]) for the trivalent inactivated vaccine and 80% [68–87%] for the trivalent live-attenuated vaccine.[50] The trivalent inactivated vaccine showed an efficacy of 60% [53–66%] in healthy adults[51] and 58% [34–73%] in the population aged >65 years.[52] The latter efficacy value was applied to all high-risk individuals using the inactivated vaccine. In the model, we assumed the efficacy of quadrivalent vaccines to be the same as that of the trivalent ones reported in the meta-analyses.[53-55] The duration of vaccination-acquired immunity is known to wane quickly after vaccination with an inactivated vaccine;[56,57] consequently, all QIV-acquired immunity was assumed to be lost after one influenza season. Immunity acquired by live-attenuated vaccination can last at least until the following season: according to an Asian study, 70% of the vaccinees who were successfully immunised in the first year with a live-attenuated vaccine were also protected in the second year against matched strains without re-vaccination.[58] Accordingly, we assumed that 30% of the immunity acquired by QLAIV vaccination was lost at the end of the first influenza season, whereas the remaining part was lost after the second season (Table 1).

### Model Outcomes

In our model, the impact of the evaluated versus the current vaccination strategy was measured in terms of reduction of symptomatic influenza cases. The cases were cumulated over the 10-year evaluation period and expressed as number of cases per 100 000 person-years, which was either calculated separately for each country or cumulated over all 14 countries. The number of symptomatic influenza cases was estimated in the total population, and separately in the subgroup of children aged 2–17 years (targeted population, direct and indirect effects) and in adults aged ≥18 years (indirect effect).

### Sensitivity and Scenario Analyses

Vaccination coverage rates with QLAIV of 25% and 75% of the 2–17 year age group were tested in two scenario analyses.

A tornado diagram was produced to show the impact of univariate variations of key parameters on the annual number of averted cases of symptomatic influenza. The included parameters were basic reproduction number $R_0$ (±10% around base case value), QLAIV efficacy of 59% in those aged 7–17 years (ie, assuming the efficacy of QIV), duration of naturally acquired immunity (±3 years around base case), duration of QLAIV-induced immunity (assuming 100% immunity loss after one season as with QIV), preferential re-vaccination factor (no increased probability or twice the base case value) and time horizon (±5 years).

### Correlation Analyses

Correlations of the influenza incidence in each country with country-specific parameters were investigated, using Spearman's correlation coefficient. These parameters include demographic factors, contact patterns, current influenza vaccination coverage, and basic reproduction number $R_0$.

**Table 1.** Simulation Tool Input Values, Common to All Countries

| Parameter | Age Group (years) | Value Base Case | | Source |
|---|---|---|---|---|
| **Population with risk factors** | | | | |
| % high-risk, without severe asthma | 0–17 | 14.3% of age group | | 3, 15 |
| | 18–44 | 16.1% of age group | | 3, 15 |
| | 45–64 | 32.1% of age group | | 3, 15 |
| | >65 | 100% of age group | | |
| % high-risk, with severe asthma | 0–17 | 1.8% of age group | | 30 |
| **Natural history** | | | | |
| External infection rate | All ages | 1/1,000 per person per year, fluctuating seasonally, peak in February | | Assumption |
| Proportion of infected developing symptoms | All ages | 66.9% | | 28 |
| Mean duration of latency | All ages | 1 day | | 28 |
| Mean duration of contagiousness | All ages | 5 days | | 28 |
| Mean duration of natural immunity after infection | All ages | Influenza A: 6 years<br>Influenza B: 12 years | | 27 |
| **Vaccine efficacy estimates (all strains)** | | QIV | QLAIV | |
| | 1 y | 45% | NA | 50 |
| | 2–17 y | 59% | 80% | 50 |
| | Adults >18, low-risk | 60% | NA | 51 |
| | Adults >18, high-risk | 58% | NA | 52 |
| **Immunity duration after vaccination** | | QIV | QLAIV | |
| % immunity loss at end of 1st season | All ages | 100% | 30% (2nd season: 100%) | 58 |
| **Re-vaccination preference factor** | All ages | RR=6.0 of being vaccinated, when vaccinated in previous year* | | 49 |
| **Vaccination strategies** | | | | |
| Reference strategy | | Current coverage, per country (Table 2) | | |
| Evaluated strategy | <2 low-risk | unchanged | 0% | |
| | 2–17 low-risk | 0% | 50% (max coverage reached in 3 years) | |
| | >18 low-risk | unchanged | 0% | |
| | 2–17 high-risk, no severe asthma | unchanged | 50% (max coverage reached in 3 years) | |
| | >18 high-risk | unchanged | 0% | |
| | >65 | unchanged | 0% | |
| | 2–17 high-risk with severe asthma | unchanged | 0% | |

NA: not applicable; QLAIV: quadrivalent live-attenuated influenza vaccine; QIV: quadrivalent inactivated vaccine; RR: relative risk
*Odds ratio 30–60 converted into relative risk using the original research[49]

**Table 2.** Simulation Tool Input Values, Country-specific

| Parameter | AU | BE | FI | FR | GE | GR | IT | LU | NL | PL | PT | SP | SW | UK |
|---|---|---|---|---|---|---|---|---|---|---|---|---|---|---|
| **Population (million)**[1] | 8.77 | 11.80 | 5.60 | 67.45 | 80.50 | 10.71 | 61.87 | 0.63 | 17.10 | 38.28 | 10.19 | 45.75 | 10.11 | 66.44 |
| % aged <18 | 17.5% | 20.7% | 19.9% | 21.8% | 15.7% | 16.9% | 16.7% | 20.8% | 19.8% | 18.1% | 16.4% | 17.2% | 21.0% | 21.5% |
| % growth 2014–25 | 6.3% | 9.8% | 5.6% | 4.8% | –0.4% | –5.8% | 3.7% | 28.8% | 3.2% | –1.1% | –4.5% | –3.1% | 9.7% | 6.9% |
| Contact matrix[2] | GE* | BE | FI | BE* | GE | IT* | IT | LU | NL | PL | IT* | IT* | FI* | UK |
| **Number of contacts**[a] | | | | | | | | | | | | | | |
| Children–Children | 1.67 | 2.73 | 3.24 | 2.91 | 1.51 | 5.57 | 5.39 | 5.22 | 5.27 | 4.27 | 1.67 | 2.73 | 3.24 | 2.91 |
| Children–Adults | 0.81 | 1.12 | 0.95 | 1.08 | 0.77 | 2.34 | 2.35 | 2.29 | 1.54 | 1.43 | 0.81 | 1.12 | 0.95 | 1.08 |
| Children–>65 | 1.03 | 1.50 | 1.24 | 1.60 | 0.98 | 1.94 | 1.92 | 1.67 | 1.37 | 1.59 | 1.03 | 1.50 | 1.24 | 1.60 |
| Adults–Adults | 0.64 | 1.18 | 0.60 | 1.14 | 0.64 | 1.82 | 1.83 | 1.38 | 0.95 | 1.13 | 0.64 | 1.18 | 0.60 | 1.14 |
| Adults–>65 | 3.99 | 5.83 | 5.33 | 5.62 | 3.91 | 8.54 | 8.52 | 8.61 | 6.91 | 8.33 | 3.99 | 5.83 | 5.33 | 5.62 |
| >65–>65 | 1.36 | 1.67 | 1.27 | 1.78 | 1.61 | 3.31 | 3.37 | 1.53 | 1.97 | 2.34 | 1.36 | 1.67 | 1.27 | 1.78 |
| Total per individual | 9.51 | 14.04 | 12.62 | 14.13 | 9.41 | 23.52 | 23.38 | 20.70 | 18.00 | 19.09 | 9.51 | 14.04 | 12.62 | 14.13 |
| **Influenza vaccination coverage (current, QIV)** | | | | | | | | | | | | | | |
| 0–17, low-risk | 6.7–9.7 | 3.5 | 5–13 | 1.6 | 4.2 | 1.4 | 2.2–4.5 | 1.5 | 1.5 | 1.4–2.4 | 1.8 | 2.3 | 1.0–2.0 | 0.5–36.9 |
| 0–17, high-risk | 18.4 | 12.6 | 24 | 17.3–42.9 | 22.6 | 18.4 | 52.4 | 4.2 | 34.8 | 7.4 | 31 | 15.7 | 8.0 | 22.3–54 |
| 18–64, low-risk | 8.9 | 11.3–18.9 | 6.2 | 6.0–21.0 | 2.0–15.0 | 6.3 | 3.4–12.0 | 4.2–13.3 | 7.3–7.4 | 3.1 | 3.0–4.9 | 2.8 | 1.1–4.2 | 1.7–7.9 |
| 18–64, high-risk | 18.4 | 22.3–32.6 | 24.0 | 31.9 | 22.6 | 18.4 | 38.0 | 4.2–13.3 | 40.6–52.5 | 7.4 | 31.0 | 18.7 | 1.1–4.2 | 48.6 |
| >65 | 38.1 | 58.0 | 34.0 | 54.0 | 50.6 | 18.4 | 62.7 | 52.4 | 74.3 | 7.4 | 45.0 | 57.0 | 45.8 | 73.4 |
| *Sources* | 33,35 | 36 | SW* | 38,63 | 39,40 | 41,64 | 7,34 | BE* | 43 | 44 | 45 | 46 | 47 | 9,48 |

AU: Austria; BE: Belgium; FI: Finland; FR: France; GE: Germany; GR: Greece; IT: Italy; LU: Luxembourg; NL: The Netherlands; PL: Poland; PT: Portugal; SP: Spain; SW: Sweden; UK: United Kingdom

[1]: EuroStat 2015 – population size calculated at the mid-point of years 2014 and 2025 population

[2]: Mossong *et al* 200816

*: Assumption, using available information from neighbouring country in absence of local data at the time of our analysis

[a]: Average number of contact per day, taking into account contact matrix and demographic structure of the country

[b]: Calibration outcome, error <1% between average target value and model output value

**Table 2.** Simulation Tool Input Values, Country-specific (continued)

| Target Endpoint | GE* | Visits | SW* | Visits | Incidence | IT* | Cases | BE* | Cases | Cases | Visits | Visits | Incidence | Cases |
|---|---|---|---|---|---|---|---|---|---|---|---|---|---|---|
| Seasons / years | | 2003–2009 | | 2005–2012 | 2006–2007 | | 2009–2012 | | 2005–2007 | 2010–2011 | 2000–2003 | 2000–2009 | 2012–2013 | 2000–2013 |
| Timeframe | | Wk 40 to Wk 20 | | Wk 1 to Wk 10 | Wk 40 to Wk 20 | | Wk 40 to Wk 20 | | Full year | Wk 40 to Wk 20 | Wk 40 to Wk 20 | Wk 1 to Wk 10 | Wk 40 to Wk 20 | Full year |
| Average value | 10.6% | 285 951 | | 1 272 857 | 10.6% | | 2 510 489 | | 410 200 | 133 167 | 55 687 | 422 978 | 10.0% | 2 662 500 |
| Min value | 7.5% | 212 195 | | 800 000 | 9.5% | | 2 330 301 | | 338 500 | NA | 29 876 | 311 668 | 7.5% | 1 893 500 |
| Max value | 22.4% | 363 880 | | 2 150 000 | 11.7% | | 2 759 959 | | 518 600 | NA | 71 282 | 592 169 | 22.4% | 3 568 500 |
| Estimated influenza infection incidence (%) | | 8.9% [6.6–11.5] | | 7.7% [4.9–12.7] | | | 6.3% [5.9–7.0] | | 3.7% [3.1–4.8] | 3.8% | 2.8% [1.5–4.6] | 3.9% [2.9–5.5] | | 12.5% [8.9–16.7] |
| Sources | GE* | 23 | SW* | 24 | 17 | IT* | 19 | BE* | 20 | 21 | 25 | 26 | 18 | 22 |
| **Basic reproduction number ($R_0$)**[b] | 1.12 | 1.18 | 1.10 | 1.28 | 1.12 | 1.05 | 1.05 | 1.10 | 0.90 | 1.05 | 0.92 | 1.02 | 1.11 | 0.96 |

AU: Austria; BE: Belgium; FI: Finland; FR: France; GE: Germany; GR: Greece; IT: Italy; LU: Luxembourg; NL: The Netherlands; PL: Poland; PT: Portugal; SP: Spain; SW: Sweden; UK: United Kingdom

[1]: EuroStat 2015 – population size calculated at the mid-point of years 2014 and 2025 population

[2]: Mossong *et al* 2008[16]

*: Assumption, using available information from neighbouring country in absence of local data at the time of our analysis

[a]: Average number of contact per day, taking into account contact matrix and demographic structure of the country

[b]: Calibration outcome, error <1% between average target value and model output value

## RESULTS

### Calibration

Model calibrations reproduced country-specific incidence targets with error rates below 1%, leading to values of the all-year average of $R_0$ which ranged from 0.90 (implying an $R_0$ peak value around Christmas of 1.29) to 1.28 (peak value 1.83) across countries (Table 2).

### Epidemiological Impact

When considering QLAIV vaccination coverage of 50% of children aged 2–17 years compared with the reference scenario, there were 2495 prevented symptomatic influenza cases per 100 000 population-years in 14 European countries. This represents a reduction of 47.6% of the symptomatic cases which occur in the reference scenario, as the absolute number of cases dropped from 228.0 to 119.4 million over 10 seasons in the 14 countries included here (absolute numbers of cases per country are shown in Supplementary material S1). Across countries, the number of symptomatic influenza cases of any age prevented by pediatric QLAIV vaccination ranged from 1366 to 3604 cases per 100 000 annually (lowest and highest values observed across 14 countries; see Table 3).

Among the targeted population of 2–17 year-old children, QLAIV vaccination prevented annually from 551 to 1555 symptomatic cases per 100 000 population across countries and 990 cases per 100 000 overall in the 14 countries that were included (Table 3). The number of pediatric cases cumulated over 10 years and 14 countries dropped from 64.1 to 21.0 million (ie, by 67.2%, with 43.1 million prevented pediatric cases in the 14 countries combined).

As a result of indirect protection, the vaccination of 2-17 year-old children with QLAIV prevented annually a range of 726–2047 cases per 100 000 population across countries (Table 3), including elderly aged >65 (pooled results for all 14 countries: 1466 prevented adult cases per 100 000 annually, of which 157.6 were prevented elderly cases per 100 000). The number of adult cases of symptomatic influenza cumulated over 10 years and 14 countries dropped from 159.7 to 95.9 million (ie, by 40.0%, with 63.8 million prevented adult cases in 14 countries).

The number of prevented cases in the non-target population (63.8 million) is 48% larger than the number of prevented cases in the target population (43.1 million).

**Table 3.** Symptomatic Influenza Cases per 100 000 Person-years by Vaccination Strategy, Country, and Target Group

| Simulation Outcome (cases per 100 000 person-years) | Symptomatic Cases Current Strategy | Symptomatic Cases Evaluated Strategy | Difference in Symptomatic Cases vs Current | % Difference in Symptomatic Cases Relative to Current |
|---|---|---|---|---|
| **Country (Total population)[a]** | | | | |
| AU (N=8.77 million) | 6456 | 3790 | –2666 | –41.3% |
| BE (N=11.80 million) | 6416 | 3791 | –2625 | –40.9% |
| FI (N=5.60 million) | 5787 | 2745 | –3043 | –52.6% |
| FR (N=67.45 million) | 8826 | 5691 | –3134 | –35.5% |
| GE (N=80.50 million) | 5509 | 3084 | –2425 | –44.0% |
| GR (N=10.71 million) | 4086 | 1547 | –2540 | –62.1% |
| IT (N=61.87 million) | 4007 | 1558 | –2450 | –61.1% |
| LU (N=0.63 million) | 6826 | 3312 | –3514 | –51.5% |
| NL (N=17.10 million) | 2405 | 1013 | –1392 | –57.9% |
| PL (N=38.28 million) | 5914 | 3224 | –2689 | –45.5% |
| PT (N=10.19 million) | 2353 | 987 | –1366 | –58.0% |
| SP (N=45.75 million) | 4214 | 1725 | –2489 | –59.1% |
| SW (N=10.11 million) | 6703 | 3099 | –3604 | –53.8% |
| UK (N=66.44 million) | 3440 | 1393 | –2047 | –59.5% |
| **Total pooled 14 countries (N=435.20 million)** | 5239 | 2744 | –2495 | –47.6% |
| **Range over 14 countries** | 2353 to 8826 | 987 to 5691 | –3604 to –1366 | –62.1% to –35.5% |

AU: Austria; BE: Belgium; FI: Finland; FR: France; GE: Germany; GR: Greece; IT: Italy; LU: Luxembourg; NL: The Netherlands; PL: Poland; PT: Portugal

SP: Spain; SW: Sweden; UK: United Kingdom

Current strategy: current vaccination coverage of high-risk individuals (country-specific rates), using QIV

Evaluated strategy: Current strategy + QLAIV in 50% of 2–17 year-old children (all countries)

[a]Exposed population in the mid-point of the evaluation period and range (2014–25).

**Table 3.** Symptomatic Influenza Cases per 100 000 Person-years by Vaccination Strategy, Country, and Target Group (continued)

| Country (children 2–17; target population)[a] | Symptomatic Cases Current Strategy | Symptomatic Cases Evaluated Strategy | Difference in Symptomatic Cases vs Current | % Difference in Symptomatic Cases Relative to Current |
|---|---|---|---|---|
| AU (N=1.37 million) | 1507 | 570 | –937 | –62.2% |
| BE (N=2.17 million) | 1641 | 612 | –1028 | –62.7% |
| FI (N=0.99 million) | 1781 | 515 | –1265 | –71.1% |
| FR (N=13.11 million) | 2464 | 1012 | –1452 | –58.9% |
| GE (N=11.32 million) | 1289 | 465 | –823 | –63.9% |
| GR (N=1.64 million) | 1307 | 322 | –985 | –75.4% |
| IT (N=9.24 million) | 1211 | 301 | –911 | –75.2% |
| LU (N=0.12 million) | 2050 | 626 | –1424 | –69.5% |
| NL (N=3.02 million) | 874 | 228 | –645 | –73.8% |
| PL (N=6.22 million) | 1556 | 489 | –1067 | –68.6% |
| PT (N=1.52 million) | 751 | 201 | –551 | –73.3% |
| SP (N=7.10 million) | 1286 | 338 | –947 | –73.7% |
| SW (N=1.88 million) | 2177 | 622 | –1555 | –71.4% |
| UK (N=12.70 million) | 1131 | 309 | –822 | –72.7% |
| **Total 14 countries (N=72.40 million)** | 1473 | 483 | –990 | –67.2% |
| **Range over 14 countries** | 751 to 2464 | 201 to 1012 | –1555 to –551 | –75.4% to –58.9% |

AU: Austria; BE: Belgium; FI: Finland; FR: France; GE: Germany; GR: Greece; IT: Italy; LU: Luxembourg; NL: The Netherlands; PL: Poland; PT: Portugal

SP: Spain; SW: Sweden; UK: United Kingdom

Current strategy: current vaccination coverage of high-risk individuals (country-specific rates), using QIV

Evaluated strategy: Current strategy + QLAIV in 50% of 2–17 year-old children (all countries)

[a]Exposed population in the mid-point of the evaluation period and range (2014–25).

| Country (Adult 18+; non-target population)[a] | Symptomatic Cases Current Strategy | Symptomatic Cases Evaluated Strategy | Difference in Symptomatic Cases vs Current | % Difference in Symptomatic Cases Relative to Current |
|---|---|---|---|---|
| AU (N=7.24 million) | 4789 | 3122 | –1667 | –34.8% |
| BE (N=9.35 million) | 4647 | 3097 | –1550 | –33.4% |
| FI (N=4.49 million) | 3881 | 2165 | –1716 | –44.2% |
| FR (N=52.73 million) | 6159 | 4539 | –1619 | –26.3% |
| GE (N=67.86 million) | 4098 | 2548 | –1551 | –37.8% |
| GR (N=8.89 million) | 2735 | 1204 | –1531 | –56.0% |
| IT (N=51.56 million) | 2744 | 1233 | –1512 | –55.1% |
| LU (N=0.50 million) | 4680 | 2633 | –2047 | –43.7% |
| NL (N=13.72 million) | 1490 | 764 | –726 | –48.7% |
| PL (N=31.35 million) | 4299 | 2702 | –1597 | –37.2% |
| PT (N=8.51 million) | 1577 | 773 | –804 | –51.0% |
| SP (N=37.88 million) | 2876 | 1360 | –1516 | –52.7% |
| SW (N=7.99 million) | 4365 | 2396 | –1969 | –45.1% |
| UK (N=52.13 million) | 2251 | 1056 | –1196 | –53.1% |
| **Total 14 countries (N=354.22 million)** | 3670 | 2204 | –466 | –40.0% |
| **Range over 14 countries** | 1490 to 6159 | 764 to 4539 | –2047 to –726 | –56.0% to –26.3% |

AU: Austria; BE: Belgium; FI: Finland; FR: France; GE: Germany; GR: Greece; IT: Italy; LU: Luxembourg; NL: The Netherlands; PL: Poland; PT: Portugal

SP: Spain; SW: Sweden; UK: United Kingdom

Current strategy: current vaccination coverage of high-risk individuals (country-specific rates), using QIV

Evaluated strategy: Current strategy + QLAIV in 50% of 2–17 year-old children (all countries)

[a]Exposed population in the mid-point of the evaluation period and range (2014–25).

## Sensitivity Analyses

Based on the univariate sensitivity analyses, the factors having the largest impact on the number of prevented influenza cases were the duration of natural immunity after influenza A and the basic reproduction number $R_0$. A longer duration of naturally acquired immunity yielded fewer prevented cases (Figure 1a), both in the target (Figure 1b) and the non-target (Figure 1c) population. Using variations of ±10% around the all-year average $R_0$ in each country led to 20% more (3007 cases per 100 000 person-years) and 27% less (1824 cases per 100 000 person-years) averted symptomatic cases, respectively, compared to the base case outcome (2495 per 100 000 person-years). The number of annually prevented cases decreased to 2176 per 100 000 (–41.5% vs. current strategy; base case –47.6%) when assuming a QLAIV efficacy of 59% in 7–17 year-old children, and to 2127 per 100 000 (–40.5%) when assuming that all QLAIV-induced immunity is lost after one season (Figure 1a). A marked direct and indirect protection was found in each evaluated scenario compared to the current vaccinaton strategy using QIV (Figure 1).

The cumulated prevented symptomatic cases in the total population increased from 52.5 million after 5 years (2414 per 100 000 person-years) to 155.7 million after 15 years (2385 per 100 000 person-years).

**Figure 1.** Univariate Sensitivity Analysis, Impact on Prevented Symptomatic Cases (pooled 14 EU countries)
*1a. Impact in Total Population*

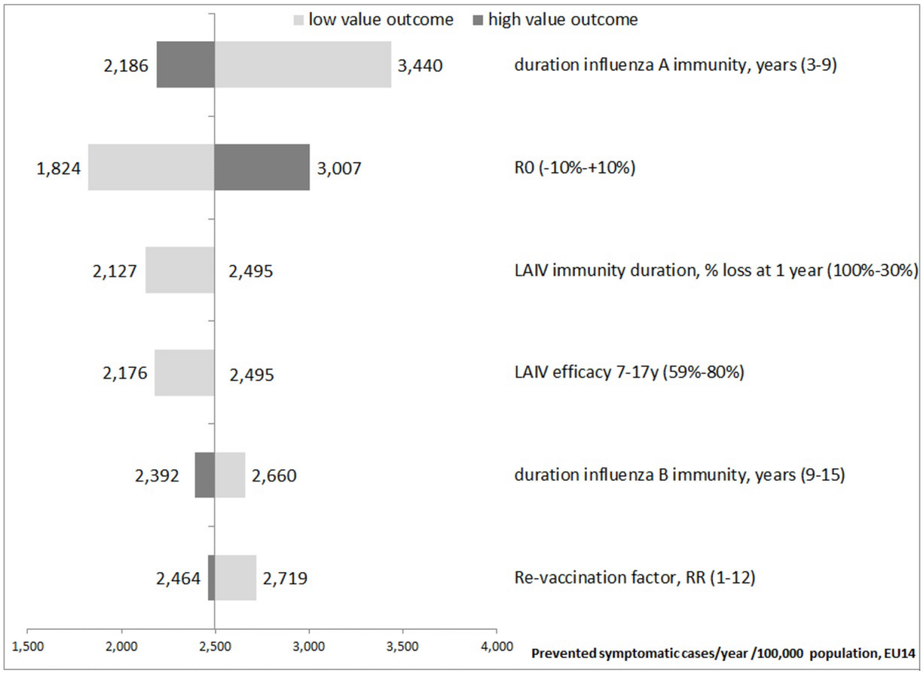

LAIV: live-attenuated influenza vaccine; $R_0$: basic reproduction number; RR: relative risk; VE: vaccine efficacy

Legend: The tornado charts in Figure 1 were obtained by changing the value of one parameter at a time and calculating the corresponding number of prevented symptomatic cases per 100 000 person-years in the total population (Fig. 1a), in the target population (children aged 2–17 years; Fig. 1b) and in the adult population (aged >18 years; Fig. 1c). The results obtained with the lower (respectively higher) tested value are shown in light grey (respectively dark grey).

The vertical axes indicate the number of prevented cases in the base case analysis: 2495 cases of any age (Fig. 1a), 990 pediatric cases (Fig. 1b), and 1466 adult cases (Fig. 1c) per 100 000 person-years.

The analysis highlights that the duration of naturally acquired immunity after influenza A infection (base case 6 years) and the basic reproduction number $R_0$ have the highest impact on the results, both in the target (children) and the non-target (adult) populations.

*1b. Impact in Children Aged 2–17 years (target population)*

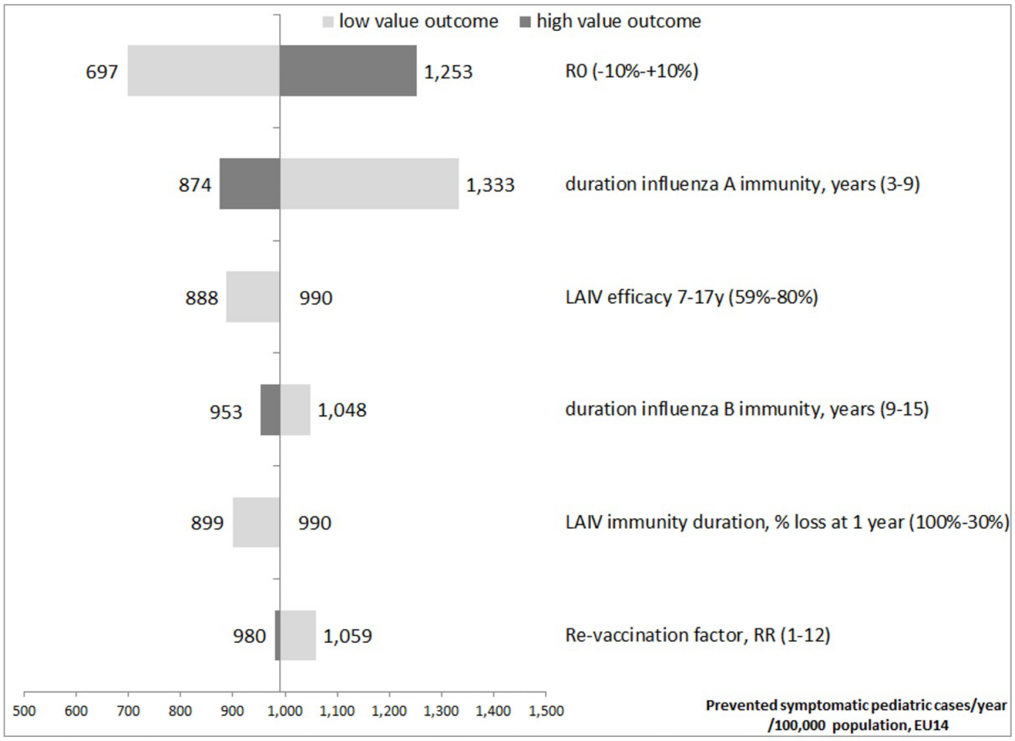

LAIV: live-attenuated influenza vaccine; $R_0$: basic reproduction number; RR: relative risk; VE: vaccine efficacy

*1c. Impact in Adults 18+ (non-target population)*

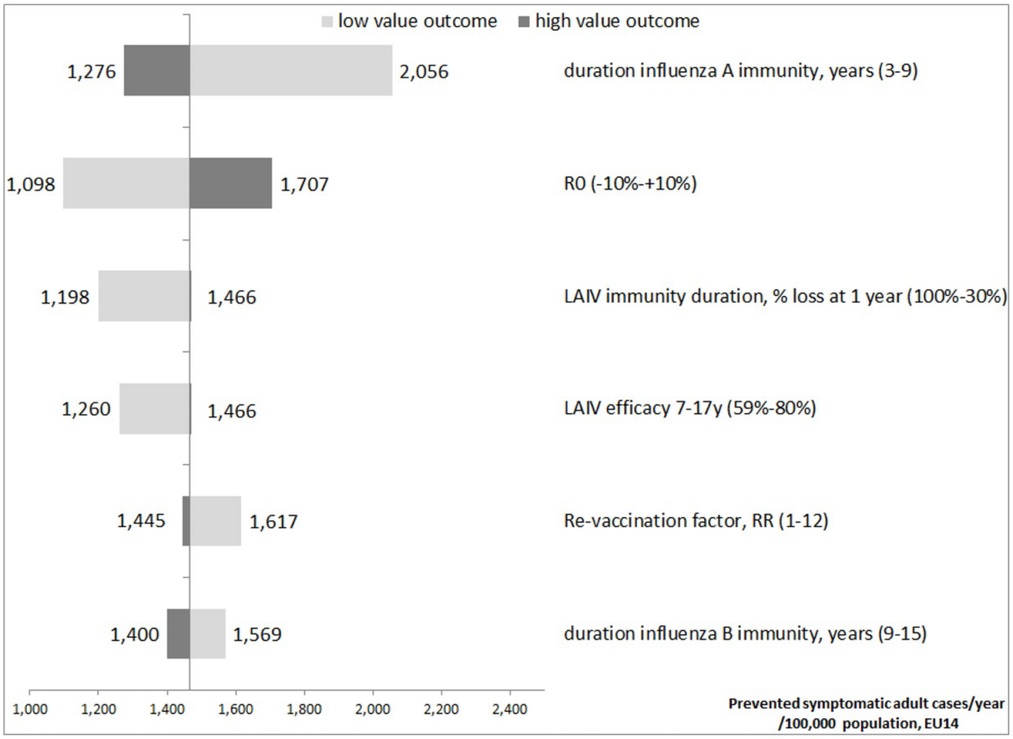

LAIV: live-attenuated influenza vaccine; $R_0$: basic reproduction number; RR: relative risk; VE: vaccine efficacy

## Scenario Analyses

When simulating a QLAIV coverage of 2–17 year-old children from 25% to 75%, the 10-year prevented cases ranged from 1532 to 3082 per 100 000 person-years, the protection effect ranged from 647 to 1168 per 100 000 person-years in the targeted population, and from 861 to 1864 per 100 000 person-years in the non-target population. With each tested coverage, the number of indirectly prevented cases exceeded the number of directly prevented cases in the target population (Figure 2).

**Figure 2.** Impact of QLAIV Coverage Rate on Symptomatic Case Reduction (pooled 14 EU countries)

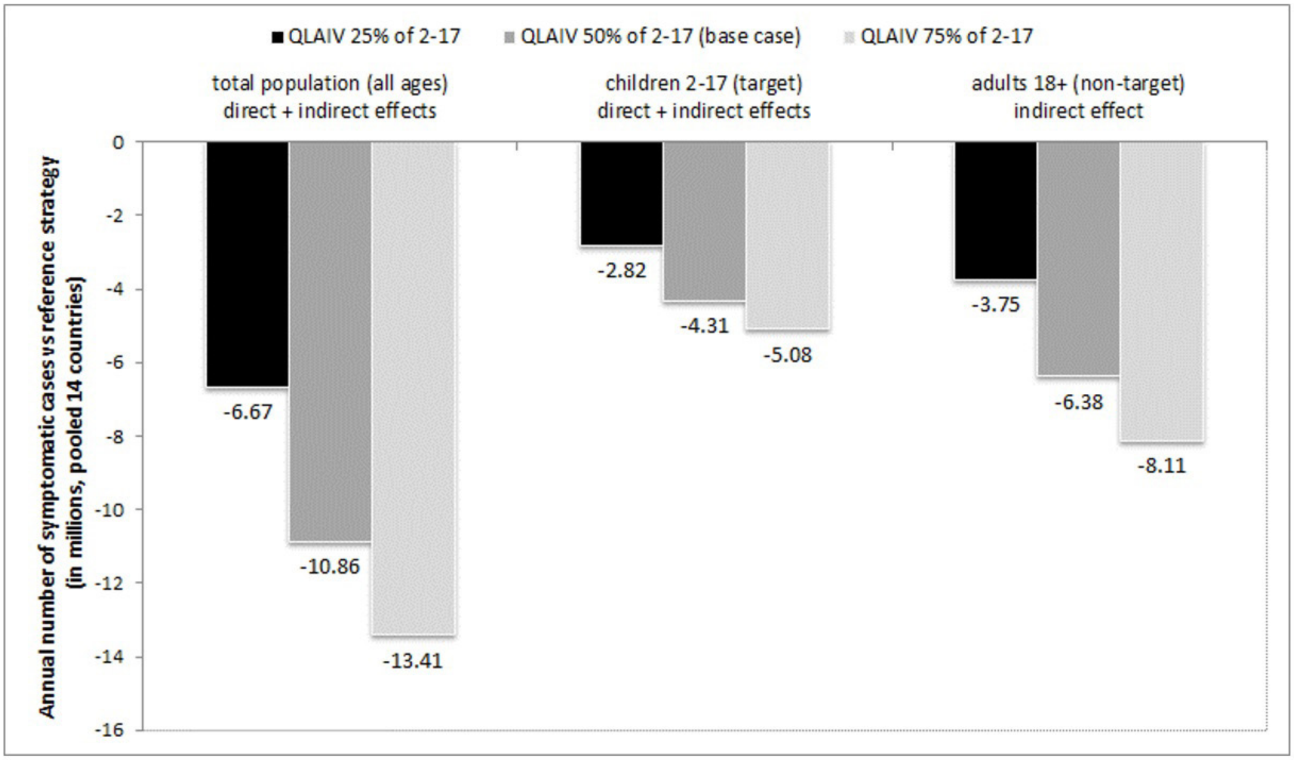

Legend: In general, the QLAIV vaccination started with the pre-existing vaccination coverage and was increased in three annual steps until the goal coverage of 25%, 50%, or 75% was reached. Subpopulations which already exceeded the vaccination goal only switched to QLAIV without modifying their vaccination coverage:

Italy, 2–17 year high-risk (52.4%)

The Netherlands, 2–17 year high-risk (34.8%)

UK, 2 year low-risk 38.1%, 3 year low-risk (40.7%), 4 year low-risk (31.9%), 2 year high-risk (53.7%), 3 year high-risk (56.4%), 4 year high-risk (52.3%), 15–17 year high-risk (47.3%).

## Correlation Analyses

The country-specific parameters which most strongly correlated with the current number of symptomatic cases per 100 000 person-years (as estimated by the model) were $R_0$ (correlation coefficient 0.82 and 95% confidence interval [0.51; 0.94]), the number of contacts between individuals aged ≥65 years (–0.62 [–0.86; –0.13]), the percentage of population growth from 2014 to 2025 (0.56 [0.05; 0.84]), the number of contacts between children and individuals aged ≥65 years (–0.54 [–0.83; –0.01]), and the current vaccination coverage rate of high-risk adults (–0.53 [–0.83; 0.00]). Results of the correlation analysis are presented in Table 4.

**Table 4.** Correlation Analysis of Country-specific Parameters with Number of Symptomatic Cases per 100 000 Person-years

| Country-specific Parameter | Spearman Correlation Coefficient | 95% Confidence Interval |
|---|---|---|
| Basic reproduction number $R_0$ | 0.82 | 0.51; 0.94 |
| Ratio Aged >65 / Aged 0–17 | –0.51 | –0.82; 0.03 |
| % Population growth 2014–25 | 0.56 | 0.05; 0.84 |
| Number of contacts: | | |
| Children (0–17) with Children | –0.54 | –0.83; –0.01 |
| Children with Adults (18–64) | –0.48 | –0.81; 0.07 |
| Children with >65 | –0.26 | –0.69; 0.32 |
| Adults with Adults | –0.31 | –0.72; 0.27 |
| Adults with >65 | –0.20 | –0.66; 0.37 |
| >65 with >65 | –0.62 | –0.86; –0.13 |
| QIV coverage: | | |
| For high-risk adults (18–64) | –0.53 | –0.83; 0.00 |
| For >65 | –0.25 | –0.69; 0.32 |

Analysis based on the number of symptomatic cases in the reference scenario of 14 countries.

A positive correlation coefficient indicates that the number of symptomatic cases per 100 000 person-years and the tested factor tends to vary in the same direction (eg, number of cases increases when $R_0$ increases).

A negative correlation coefficient indicates that the number of symptomatic cases per 100 000 person-years and the tested factor tends to vary in opposite directions (eg, number of cases increases when the QIV coverage of high-risk adults decreases).

A 95% confidence interval not including zero indicates that the correlation is statistically significant (p-value <0.05).

## DISCUSSION

Our results demonstrate large epidemiological benefits in Europe comprised of both direct and indirect elements if healthy children aged 2–17 years are vaccinated with QLAIV and a 50% vaccine coverage rate is reached. Results were robust and conclusions remained unchanged across a range of univariate sensitivity and scenario analyses. The most influential parameters are the duration of naturally acquired immunity after influenza A infection and the basic reproduction number $R_0$.

The magnitude of our results is moderate compared to the highly positive results of UK modelling studies which reported that up to 84% of cases can be averted (as compared to the current policy over multiple seasons) in the total population when vaccinating 50% of the children with LAIV.[8,9] This difference is partly due to an effect which can also be seen in our simulations: in the first years after introducing a new vaccination campaign, the annual incidence reaches a minimum before (a few years later) a new, slightly higher quasi-equilibrium establishes. This effect (which has been termed "honeymoon period"[59]) is caused by a combination of natural immunity aquired in earlier years while transmission was still high, and the newly increased level of vaccination-derived immunity. Our approach of increasing the vaccination coverage over 3 years and evaluating a time period of 10 years, therefore, should come to somewhat more moderate results than a scenario in which the vaccination coverage may be abruptly increased with evaluation of benefit shortly thereafter. While dynamic transmission models are difficult to compare given the range of required assumptions and sophisticated programming techniques, the different published models to date do highlight a clinical benefit of pediatric LAIV vaccination. The countries' specificities, including contact patterns, demographic changes and influenza incidence targets, also account for different magnitudes of results as highlighted in the correlation analysis. These local parameters were found to strongly influence the success of vaccination.[60] Our 14 countries were mainly selected based on their inclusion in the Polymod study as this study provided contact data suitable for modelization;[16] further EU countries were added to increase the representativeness of our study at the European level. Our purpose was to present an overall EU picture of the QLAIV vaccination effect rather than a between-countries comparison. The calibration process ensures that local targets are reached by adjusting the basic reproduction number $R_0$. However, given the independence of data sources used, the countries having the highest incidence targets are not necessarily those with, for example, the largest numbers of between-individual contacts. This explains why the modelled results can lead to counter-intuitive correlations with local factors (see Table 4) and why country differences can be expected.

Our study focused on symptomatic influenza cases, as the main purpose was to understand the magnitude of direct and indirect protection across a range of sensitivity analyses and country-specific features. Based on the prevented symptomatic cases, it is possible to extrapolate the modelled clinical benefit in each country to an impact in terms of medical resources used, which is relevant for decision makers. Assuming that 33% of symptomatic cases lead to a primary care consultation and 1% lead to an hospital admission[61], our findings translate into 35.8 million prevented consultations and 1.09 million prevented hospitalisations cumulated over 10 years and 14 European countries. Ten QLAIV vaccinations are needed to prevent one influenza consultation, and 300 to prevent one hospitalisation. This finding is in line with real-life data published for season 2014–2015 in the UK where 16 QLAIV vaccinations were needed to prevent one consultation and 317 to prevent one hospitalisation, when vaccinating 56.8% of primary school age children.[7] In recent seasons, however, lower QLAIV effectiveness against influenza A/H1N1pdm09 was observed[62], which could not be included in our model at the time of our analyses. This trend is expected to impact the direct and indirect benefits described in this article, to an extent which remains to be evaluated. Comparison of modelled and real-life outcomes from the UK programme will be the topic of subsequent research using the same European dynamic transmission model.

As observed data are still scarce, modelling studies highlight a positive impact of a pediatric immunisation programme against influenza, across a range of demographic features, contact patterns, current vaccination coverage, and local influenza incidence. Our analyses therefore inform policy makers of the benefit of pediatric QLAIV vaccination in Europe, not only in the targeted population of vaccinated children but also in terms of indirect protection against influenza-related outcomes in the general population.

## FINANCIAL SUPPORT

This study was funded by an unrestricted grant from AstraZeneca.

## CONFLICT OF INTEREST

JH, RL, and SDSM are or were employees of AstraZeneca at the time of the study. LG is an employee of IMS Health which has received consulting fees from AstraZeneca. MS is an employee and shareholder of ExploSYS GmbH, which has received payments from Epimos GmbH, a contract research and consulting institute, which has received research support and consulting fees from AstraZeneca. ME is a partner and shareholder of the contract research and consulting institute Epimos GmbH, which has received consulting fees and research support from AstraZeneca, Novartis, and GlaxoSmithKline. CWO has received grants for congresses and honoraria for conferences and meetings from AstraZeneca, GlaxoSmithKline, Novartis, Pfizer, Sanofi-Pasteur, and Sanofi-Pasteur MSD.

## AUTHORSHIP

ME conceptualised the study. ME carried out the simulations and interpreted the results. MS designed and developed the simulation tool and provided technical support. LG provided local data inputs, analysed the simulation results, and drafted the manuscript. CWO, JH, RL and SDSM provided expertise and guidance on data input and assumptions. All authors critically appraised, corrected and approved the manuscript before submission.
